# Supplementary material for: Pharmacological Ascorbate Induces Transient Hypoxia Sensitizing Pancreatic Ductal Adenocarcinoma to a Hypoxia Activated Prodrug
Source: bioRxiv. 2024 May 15:2024.05.13.593896. Preprint. [Version 1] doi: 10.1101/2024.05.13.593896 (PMC12478372; doi:10.1101/2024.05.13.593896)
Supplement: Supplement 1 [file NIHPP2024.05.13.593896v1-supplement-1.pdf]

bioRxiv preprint doi: https://doi.org/10.1101/2024.05.13.593896; this version posted May 15, 2024. The copyright holder for this preprint (which was not certified by peer review) is the author/funder. This article is a US Government work. It is not subject to copyright under 17 USC 105 and is also made available for use under a CC0 license.

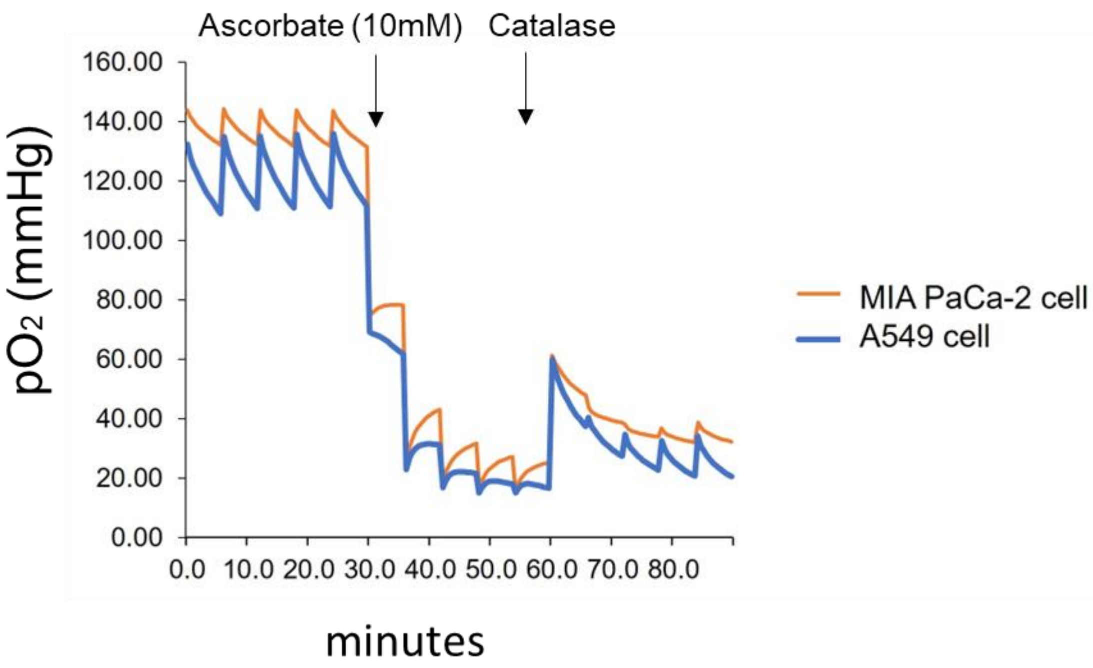

Supplementary Figure 1: Representative oxygen profile uncorrected for diffusion

bioRxiv preprint doi: https://doi.org/10.1101/2024.05.13.593896; this version posted May 15, 2024. The copyright holder for this preprint (which was not certified by peer review) is the author/funder. This article is a US Government work. It is not subject to copyright under 17 USC 105 and is also made available for use under a CC0 license.

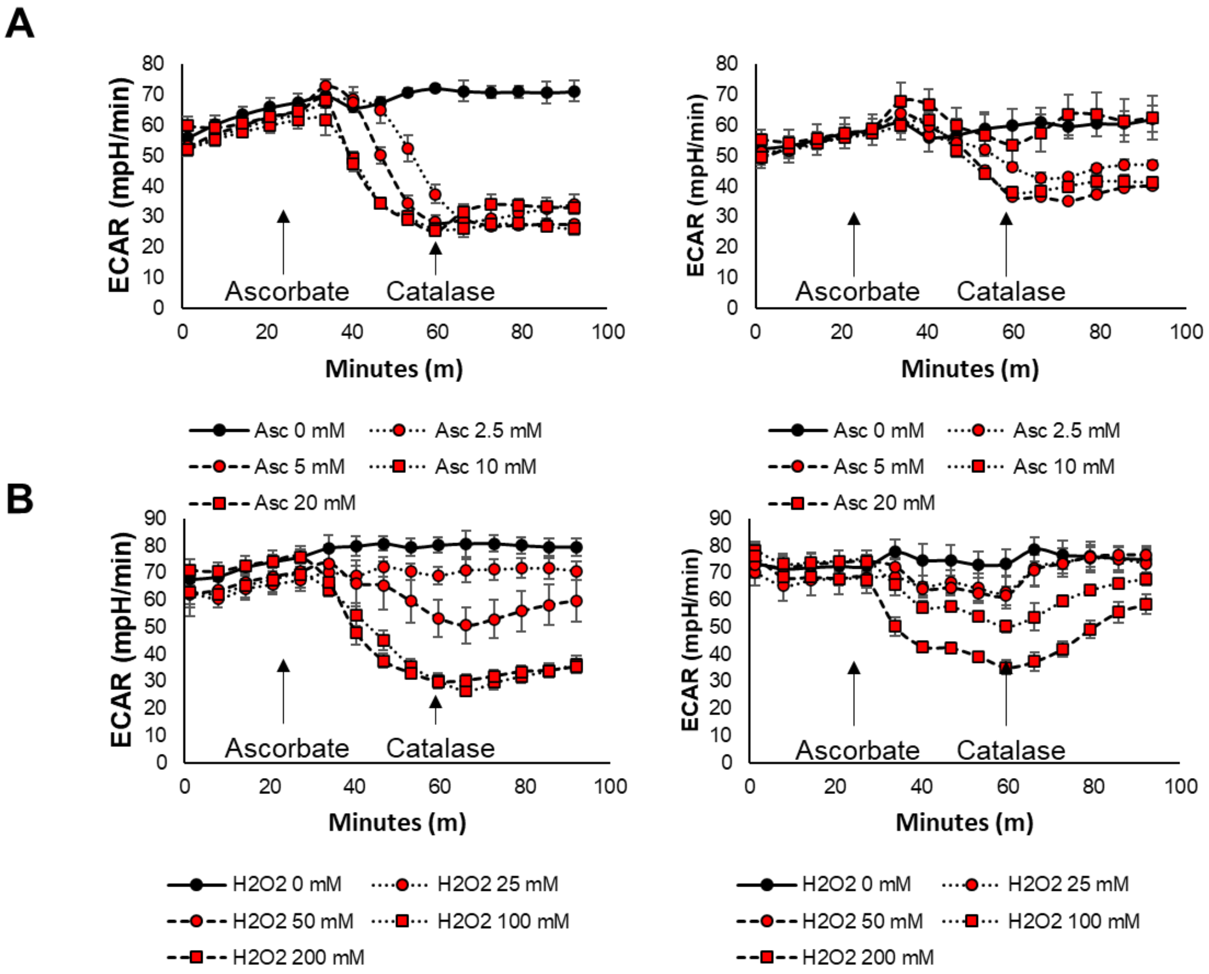

Supplementary Figure 2 Extracellular Acidification Rates (Rates) for the OCR plotted in Figure 3. Add seconds to x axis or indicate x axis is in seconds.
